# The effectiveness of a proven chronic disease prevention and screening intervention in diverse and remote primary care settings: an implementation study on the BETTER 2 Program

**DOI:** 10.3399/bjgpopen19X101656

**Published:** 2019-09-18

**Authors:** Kris Aubrey-Bassler, Carolina Fernandes, Carla Penney, Richard Cullen, Christopher Meaney, Nicolette Sopcak, Denise Campbell-Scherer, Rahim Moineddin, Julia Baxter, Paul Krueger, Margo Wilson, Andrea Pike, Eva Grunfeld, Donna Manca

**Affiliations:** 1 Director, Primary Healthcare Research Unit, Memorial University of Newfoundland, St. John’s, Canada; 2 Lead Coordinator, Department of Family Medicine, Faculty of Medicine & Dentistry, University of Alberta, Edmonton, Canada; 3 Research Coordinator, Primary Healthcare Research Unit, Memorial University of Newfoundland, St. John's, Canada; 4 Research Coordinator, Primary Healthcare Research Unit, Memorial University of Newfoundland, St. John's, Canada; 5 Biostatistician, Department of Family and Community Medicine, Faculty of Medicine, University of Toronto, Toronto, Canada; 6 Qualitative Research Lead, Department of Family Medicine, Faculty of Medicine & Dentistry, University of Alberta, Edmonton, Canada; 7 Professor, Department of Family Medicine, Faculty of Medicine & Dentistry, University of Alberta, Edmonton, Canada; 8 Professor, Department of Family & Community Medicine, Faculty of Medicine, University of Toronto, Toronto, Canada; 9 Research Program Coordinator, Department of Family & Community Medicine, Faculty of Medicine, University of Toronto, Toronto, Canada; 10 Associate Professor, Department of Family & Community Medicine, Faculty of Medicine, University of Toronto, Toronto, Canada; 11 Clinical Assistant Professor of Emergency Medicine, Discipline of Emergency Medicine, Faculty of Medicine, Memorial University of Newfoundland, St. John’s, Canada; 12 Research Manager, Primary Healthcare Research Unit, Memorial University of Newfoundland, St. John's, Canada; 13 Director, Knowledge Translation Research, Health Services Research Program, Ontario Institute for Cancer Research, Toronto, Canada; 14 Professor and Director of Research, Department of Family Medicine, Faculty of Medicine & Dentistry, University of Alberta, Edmonton, Canada

**Keywords:** chronic disease, primary prevention, early detection of cancer, disease management, general practice, primary care

## Abstract

**Background:**

The **B**uilding on **E**xisting **T**ools **t**o Improv**e** Ch**r**onic Disease Prevention and Screening in Primary Care (BETTER) randomised control trial (RCT) showed that the BETTER Program improved chronic disease prevention and screening (CDPS) by 32.5% in urban team-based primary care clinics.

**Aim:**

To evaluate outcomes from implementation of BETTER in diverse clinical settings.

**Design & setting:**

An implementation study was undertaken to apply the CDPS intervention from the BETTER trial to diverse settings in BETTER 2. Patients aged 40–65 years were invited to enrol in the study from three clinics in Newfoundland and Labrador, Canada.

**Method:**

At baseline, eligibility for 27 CDPS actions (for example, cancer, diabetes and hypertension screening, lifestyle) was determined. Patients then met with a trained provider and prioritised goals to address their eligible CDPS actions. Providers received training in behaviour change theory and practice. Descriptive analysis of clinical outcomes and success factors were reported.

**Results:**

A total of 154 patients (119 female and 35 male) had a baseline visit; 106 had complete outcome assessments, and the remainder had partial outcome assessments. At baseline, patients were eligible for a mean of 12.3 CDPS actions and achieved a mean of 6.0 (49%, 95% confidence intervals [CI] = 24% to 74%) at 6-month follow-up, including reduced hypertension (86% of eligible patients, 95% CI = 67% to 96%), weight control (51% of eligible patients, 95% CI = 42% to 60%), and smoking cessation (36% of eligible patients, 95% CI = 17% to 59%). Male, highly educated, and lower income individuals achieved a higher proportion of CDPS manoeuvers than their counterparts.

**Conclusion:**

Clinical outcomes from this implementation study were comparable with those of the prior BETTER RCT, providing support for the BETTER Program as an effective approach to CDPS in more diverse general practice settings.

## How this fits in

Family physicians lack the time and resources to effectively address all CDPS guidelines during routine patient visits. In the BETTER pragmatic cluster RCT, a novel approach to CDPS led by a non-physician provider improved the achievement of CDPS activities by more than 30% compared with usual care in the general practice setting. In this study, BETTER 2, the adaptation of the intervention was explored in more diverse clinical settings. This article presents the quantitative clinical outcomes from BETTER 2.

## Introduction

The prevalence of chronic disease is increasing at a dramatic rate, and a large proportion of these diseases are avoidable, yet health systems continue to focus overwhelmingly on the treatment rather than prevention of these diseases.^1^ Evidence suggests that prevention programmes can be successful, but widespread implementation has been lacking in part because primary care physicians lack the time and resources to do so.^2^ In addition, with 45% of people having multiple chronic conditions, primary care providers need effective tools to be able to address this complexity.^3^ Unfortunately, evidence-based guidelines are often focused on only one specific risk factor or condition, which makes it difficult for providers to be able to assess and address the unique needs of their patients.^4,5^ What is needed is a comprehensive, evidence-based approach to CDPS that bridges the gap between CDPS and practice.^5^ The BETTER Program meets these criteria (Box 1). The clinical effectiveness of this approach was shown in the BETTER pragmatic cluster RCT, hereafter referred to as the BETTER trial.^6^ The patient-level intervention component of the trial improved CDPS by 32.5% relative to usual care. This component was led by a 'prevention practitioner' (PP), who met with patients within the practice setting. The PP used the BETTER trial tools and a process of shared decision-making to create a tailored 'prevention prescription' for each participant. PPs had professional backgrounds as nurse practitioners, registered nurses, licensed practical nurses, or dieticians, and they developed additional, specialised skills in CDPS and behaviour change in order to deliver the BETTER intervention. Qualitative^7^ and cost-effectiveness^6^ analyses of the PP intervention for the BETTER trial showed positive results, providing strong support for further exploration of the PP intervention as a viable approach to CDPS in general practice.

Box 1 **The BETTER 2 tools (available at http://www.better-program.ca/practice-resources/)**

**Table:** 

**Tool**	**Description**
The BETTER Health Survey	A survey including validated[Table-fn T1_FN1] ^a[Table-fn T1_FN1]^ tools to capture alcohol use, diet, physical activity, smoking, family history, and demographic information. Designed to be completed by patients before the prevention visit.
The BETTER Care Map	Combines all the evidence on CDPS actions, providing their associated targets and corresponding care paths. Designed to be used by the PP to assist with decision-making.
The Spaghetti Diagram	Previously published diagram illustrating the interrelation of various lifestyle factors and disease risk. Designed to be used by the PP to assist with patient education.
The Bubble Diagram	A visual representation of the BETTER algorithm containing sex-specific prevention and screening targets. Designed to be used by the PP to assist with patient education about disease risk factors.
The BETTER Prevention Prescription	A summary of the patient’s risk for chronic disease and their discussion with the PP. Contains the Goals Sheet on the reverse. Designed to be used by the PP for patient information.
The BETTER Goals Sheet	Contains three prevention goals set by the patient with the support and guidance of the PP. Designed as a patient motivation and planning tool.

CDPS = chronic disease prevention and screening. PP = prevention practitioner.

^a^where possible

The BETTER trial sample was predominantly white, female, well-educated, with mid-to-high household income, living in two Canadian urban centres.^6^ The objective of the current study, BETTER 2, was to adapt and study the implementation of the successful CDPS intervention from the BETTER trial in rural, remote, and underserved populations from Newfoundland and Labrador, Canada. This article reports on the clinical outcomes from BETTER 2. A description of tool adaptation^8^ and an assessment of participant satisfaction^9^ are published elsewhere.

## Method

### Setting

Using the existing network of fellow clinicians and administrators in the regional health authorities from the BETTER Program, three primary care practices were identified in Newfoundland and Labrador serving populations that differed significantly from those enrolled in the BETTER trial.^6^ The BETTER trial population reflected primarily urban, middle-class individuals with a mid-to-high household income; therefore, BETTER 2 was implemented in more diverse settings, including rural and remote areas, to capture individuals with lower income and/or from disadvantaged settings. As a result, the chosen settings were: 1) a primary care clinic attached to a hospital in an remote town serving a proportionately large indigenous population; 2) a rural shared-care family physician and nurse practitioner practice serving two small towns, approximately 90 minutes from an urban centre; and 3) an urban academic family practice that cared for a high proportion of immigrants and refugees in the provincial capital city. A PP identified by, or appointed to, each site was trained by the BETTER Program team to deliver the intervention using the toolkit developed for the BETTER 2 study^8^ and brief action planning (see below for description).^10^

### Patients and recruitment

Patients aged 40–65 years were invited to enroll in the programme using a combination of waiting-room posters, mailed flyers, clinician referral, local newspaper advertisements, and mail-out invitations. Interested patients contacted the participating clinic to schedule their appointment. At the first visit, the PP provided the patient with the written consent form, which was explained by the PP and signed by the patient after addressing any questions the patient had. To maximise generalisability, there were minimal exclusion criteria. Patients were excluded if they were unable to give informed consent for reasons of language, literacy, or competency, and if they were not able to attend the initial PP visit in person at their primary care site (for example, because of issues with mobility).

### Design and intervention

The intervention termed the 'patient-level intervention with a prevention practitioner' was identical to that used in the BETTER trial,^6^ with the exceptions described below. The tools originally developed by the BETTER team and used for the BETTER trial intervention were updated for use in BETTER 2 ('the BETTER 2 tools')^8^ and are described in Box 1. Patients were mailed a copy of the BETTER Health Survey (further information available from the author on request) by the clinic after booking their first appointment and were asked to bring a completed copy to the visit. The original BETTER Health Survey consisted of 88 items, and included an assessment of physical activity and a dietary assessment derived from MEDFICTs, an instrument with a focus primarily on fat intake.^11,12^ The health survey was refined for use in BETTER 2 based on feedback received following the BETTER trial which suggested that it could be streamlined and reformatted to improve data capture and usability. The health survey was shortened from 88 to 69 items to better capture information on modifiable lifestyle risk factors (for example, smoking, alcohol use, diet and nutrition, and physical activity) as well as assessments of the patient’s readiness to change. In particular, the changes included a general practice activity questionnaire (GPPAQ) to better capture and determine the patient's level of activity; a validated tool for dietary assessment and intervention called 'starting the conversation' was added to provide insight into patients’ eating behaviour and information on how diet can be improved; and alcohol consumption was quantitatively measured using guidelines according to the National Institute on Alcohol Abuse and Alcoholism.^8^ The survey also included a screening tool, the AUDIT-C, for screening and educating patients on alcohol use disorders.^8^ Participants who reported having difficulty with the survey completed it with the assistance of the PP. The PP would read the survey questions and answers to the patient who would verbally respond; if a patient was uncomfortable with answering a question, they could opt to skip the question. PPs reviewed the survey and the patient chart before meeting with the patient. The survey was designed to acquire information that is not typically recorded in the chart. Collectively, these two sources were used to learn about each patient’s overall health, including information on physical activity, smoking, alcohol consumption, diet, cancer, chronic disease, and family history. In the lifestyle section of the survey, patients also indicated their readiness to and confidence about change on these domains.

The PP then conducted a 60-minute clinical consultation about CDPS, and recorded information relevant to the patient’s chronic disease risk, including their status on screening for chronic disease and associated lifestyle factors, on a 'prevention prescription' (further information available from the author on request). Using shared decision-making and the principles of brief action planning,^10^ the PP and patient collaboratively set up to three goals to address the patient’s prevention and screening, and recorded them on the patient’s Goal Sheet (further information available from the author on request). Brief action planning is an approach that is based on motivational interviewing. It is a self-management support tool for chronic conditions, health, and wellbeing that involves a structured step-by-step process to help individuals set goals and make concrete action plans. It is structured around three core questions: question one is asked to elicit ideas for change; question two is asked to evaluate confidence; and question three is to arrange follow-up or accountability.^10^ Each PP is trained in brief action planning (provided by the Centre for Collaboration, Motivation and Innovation [CCMI]) and used these principles along with shared decision-making to help patients make realistic, attainable goals for their health. Follow-up visits to review the prevention prescription and goals were scheduled every 6 months for 12–18 months. Before the second visit, patients completed a brief version of the BETTER Health Survey and the PP reviewed the patient’s chart again to assess outcomes. Second visits were typically 15–30 minutes long and were conducted via telephone or in person, based on patient preference and convenience.

### Outcomes

The primary outcome for this study was a composite index comprised of 27 CDPS actions, including process, referral and/or treatment, and lifestyle target, and/or change items (further information available from the author on request). Actions included in the outcome were identified by the BETTER clinical working group before implementation using a comprehensive evidence synthesis of high-quality international, national, and provincial guidelines and recommendations for the chronic diseases in scope for the BETTER Program, including associated lifestyle factors;^5^ the list was updated for BETTER 2.^8^ At baseline, PPs used the BETTER Health Survey and the medical record (for example, laboratory testing, procedure reports) to determine whether the patient was eligible (that is, overdue or off-target) for each action. These items formed the list of eligible actions for that patient. The quantitative clinical study outcome was the proportion of eligible actions that were completed at 6-month follow-up. Baseline eligibility and achievement criteria for the 27 actions are available from the authors on request. The outcome for BETTER 2 was modified slightly from the BETTER trial^6^ outcome to reflect updated screening guidelines and lifestyle recommendations, and to improve clinical relevance.

### Statistical analysis

For the BETTER Program evaluation, patient and practice characteristics were summarised using means and standard deviations (SDs) for continuous variables, and counts and percentages for categorical variables. Exact binomial CIs were used to characterise variability in the achievement of individual actions from within the composite outcome. Noting that patient-level responses were nested and/or clustered within physicians in this study design, linear generalised estimating equation (GEE) models were used to investigate the impact of patient-level covariates on composite outcome. Both bivariate and multivariate linear GEE models were estimated and estimated regression coefficients, 95% CIs, and *P* values from these models have been reported. All analyses were conducted using SAS software (version 9.4).

## Results

A total of 154 patients were enrolled and attended a baseline visit with a PP. One hundred and six (69%) participants attended a follow-up visit before the study end date. The average duration from baseline to follow-up visit was 217.4 days. Medical records were reviewed to assess achievement of as many prevention and screening targets as possible for the 48 patients who did not attend a follow-up visit. Table 1 describes the demographic characteristics of the BETTER trial and BETTER 2. Participants enrolled in BETTER 2 were largely female (77%, *n* = 119) with an average age of 56 years (SD ±5.8 years). Most (67%, *n* = 103) were white but, unlike the BETTER trial, the second most common ethnic group was indigenous (18%, *n* = 28). A majority of participants had some college or university education and 55% (*n* = 84) were fully employed, with an average family or household income >$60 000 per year (CAD, 72%). The demographic characteristics of participants enrolled in BETTER 2 are similar to those who participated in the BETTER trial, with the exception of a higher recruitment rate of indigenous participants. Table 2 describes the achievement of CDPS goals. BETTER 2 patients were eligible for a mean of 12.3 CDPS actions at baseline; that is, patients were overdue or off-target for an average 12.3 evidence-based manoeuvers at the time of their first visit with the PP. At the time of the follow-up visit, patients completed an average of 6.0 (49%) of those manoeuvers for which they were eligible. The number of patients eligible for each of the 27 actions included in the composite outcome ranged from 2 to 147 (Table 3). In the adjusted analysis, men, non-smokers, lower income, and more highly educated patients improved on the composite index to a greater degree than their counterparts. See Table 4 for these and other results of the bivariate and multivariate regression analyses.

**Table 1.** Selected baseline characteristics of the BETTER trial and BETTER 2 participants

	BETTER trial^a^	BETTER 2
Characteristic	*n* = 209	Remote^b^ *n* = 57	Rural^b^ *n* = 23	Urban^b^ *n* = 74	Total*n* = 154
**Female, *n* (%)**	138 (66)	41 (72)	20 (87)	58 (78)	119 (77)
**Mean a** **ge** **,** **years (SD)**	53 (6.7)	55 (6.7)	57 (6.7)	56 (7.0)	56 (6.8)
**Ethnic** **group, *n* (%)**					
European	184 (88)	22 (39)	16 (94)	65 (88)	103 (67)
Indigenous	2 (1)	28 (49)	0 (0)	0 (0)	28 (18)
Other	23 (11)	^c^	^c^	^c^	5 (3)
**Citizenship, *n* (%)**					
Canadian	161 (77)	56 (98)	23 (100)	67 (91)	146 (95)
Other	48 (23)	^c^	^c^	7 (10)	8 (5)
**Education, *n* (%)**					
High school or lower	25 (12)	3 (5)	14 (61)	5 (7)	22 (14)
Some college/university	144 (69)	46 (81)	8 (35)	46 (62)	100 (65)
Graduate degree	40 (19)	^c^	^c^	21 (29)	29 (19)
**Employment, *n* (%)**					
Fully employed	140 (67)	34 (60)	6 (26)	44 (60)	84 (55)
Retired	10 (5)	10 (18)	7 (30)	17 (23)	34 (22)
Other	59 (28)	13 (23)	9 (39)	12 (16)	34 (22)
**Married** **or** **c** **ommon** **l** **aw, *n* (%)**	155 (74)	47 (82)	21 (91)	54 (73)	122 (79)
**Income, *n* (%)**					
<$60 000	40 (19)	7 (12)	10 (53)	19 (26)	36 (23)
$60 000–$99 999	65 (31)	17 (30)	7 (37)	15 (20)	39 (25)
≥$100 000	104 (50)	23 (40)	2 (11)	30 (41)	55 (36)
**Current** **s** **moker** **,** ***n*** **(%)**	31 (15)	10 (18)	1 (4)	6 (8)	17 (11)
**Alcohol** **c** **onsumption** **,** ***n*** **(%)**					
Never	31 (15)	8 (14)	5 (23)	13 (18)	26 (17)
Less than weekly	100 (48)	36 (63)	10 (43)	27 (37)	73 (47)
Weekly or more	77 (37)	13 (23)	7 (30)	33 (45)	53 (35)
**Exercise, *n* (%):**					
<150 minutes/week	169 (81)	39 (68)	12 (52)	33 (45)	84 (55)
≥150 minutes/week	40 (19)	18 (32)	11 (48)	41 (55)	70 (45)
**Mean** **BMI** **(SD)**	26 (5.8)	33 (6.2)	31 (4.2)	30 (5.6)	31 (5.7)
BMI ≥30, *n* (%)	52 (25)	35 (61)	13 (57)	34 (46)	82 (53)
**Mean w** **aist** **c** **irc** **,** **cm (SD)**	108 (4)	102 (14)	108 (10)	99 (13)	101 (13)
**Diabetes** **m** **ellitus** **,** ***n*** **(%)**	15 (7)	11 (19)	1 (4.8)	10 (14)	14 (9)
**Coronary** **a** **rtery** **d** **is** **ease** **,** ***n*** **(%)**	10 (5)	11 (19)	11 (48)	9 (12)	32 (21)
**Family** **h** **istory** **of…, *n* (%)**					
Breast cancer	31 (15)	^c^	^c^	^c^	9 (6)
Colorectal cancer	23 (11)	^c^	^c^	2 (1)

Circ = circumference. Dis = disease. SD = standard deviation.

^a^BETTER trial demographics for patients receiving the prevention practitioner intervention are presented for comparison purposes. ^b^See Method section for an expanded description of study sites. ^c^Suppressed for privacy reasons because of small numbers.

**Table 2.** Prevention and screening actions by study (BETTER trial, BETTER 2) and randomisation group^a^

	**BETTER trial**	**BETTER 2**
**Control**	**PP**	
Patients*, n*	183	209	154
Eligible actions, mean (SD)^b^	9.1 (3.4)	8.9 (3.2)	12.3 (2.8)
Achieved actions, mean (SD)^c^	1.9 (1.8)	4.7 (2.7)	6.0 (3.1)
Actions achieved, % (SD)	21.0 (17.5)	53.6 (26.0)	49.3 (25.0)

PP = prevention practitioner.

^a^Randomisation group applies only to the BETTER trial.

^b^The number of actions patients were eligible to improve at baseline.

^c^The number of eligible actions achieved at follow-up.

**Table 3.** Eligibility and achievement of individual prevention and screening actions

Prevention and screening actions^a^	Eligible^b^	Achieved^c^
*n*	%	*n*	%	95% CI
Screening					
1. FBS or HbA1c monitoring (*n* = 145)	3	2.1	1	33.3	0.8 to 90.6
2. BP monitor (*n* = 153)	6	3.9	1	16.7	0.4 to 64.1
3. Cervical cancer screen (*n* = 90)	8	8.9	6	75	34.9 to 96.8
4. Breast cancer screen (*n* = 115)	16	13.9	12	75	47.6 to 92.7
5. BP screen (*n* = 153)	26	17.0	15	57.7	36.9 to 76.7
6. CRC screen (*n* = 154)	36	23.4	14	38.9	23.1 to 56.5
7. LDL measured (*n* = 153)	40	26.1	25	62.5	45.8 to 77.3
8. FBS or HbA1c screen (*n* = 154)	45	29.2	20	44.4	29.6 to 60.0
9. Smoking screen (*n* = 154)	65	42.2	24	36.9	25.3 to 49.8
10. Alcohol use screen (*n* = 154)	99	64.3	48	48.5	38.3 to 58.8
11. Physical activity screen (*n* = 154)	104	67.5	63	60.6	50.5 to 70.0
12. Nutrition screen (*n* = 154)	129	83.8	53	41.1	32.5 to 50.1
13. BMI screen (*n* = 154)	131	85.1	108	82.4	74.8 to 88.5
14. Waist circumference recorded (*n* = 154)	147	95.5	123	83.7	76.7 to 89.3
**Treatment** **i** **nitiation** **or** **r** **eferral**					
15. Cholesterol treatment (*n* = 121)	2	1.7	2	100	15.8 to 100.0
16. Referral smoking cessation (*n* = 154)	22	14.3	5	22.7	7.8 to 45.4
17. Referral alcohol cessation (*n* = 154)	104	67.5	4	3.9	1.1 to 9.6
18. Referral nutrition (*n* = 154)	123	79.9	44	35.8	27.3 to 44.9
19. Referral physical activity (*n* = 153)	130	85.0	36	27.7	20.2 to 36.2
20. Referral weight control (*n* = 151)	132	87.4	58	43.9	35.3 to 52.8
**Risk** **m** **odification**					
21. LDL improvement (*n* = 121)	18	14.9	11	61.1	35.8 to 82.7
22. Smoking cessation (*n* = 154)	22	14.3	8	36.4	17.2 to 59.3
23. Hypertension control (*n* = 153)	29	19.0	24	85.7	67.3 to 96.0
24. At risk alcohol improvement (*n* = 154)	79	51.3	32	60.4	46.0 to 73.4
25. Diet score improvement (*n* = 154)	123	79.9	54	62.1	51.0 to 72.3
26. Physical activity improvement (*n* = 153)	130	85.0	61	67	56.4 to 76.5
27. Overweight stabilisation (*n* = 151)	132	87.4	67	50.8	41.9 to 59.6

BMI = body mass index. BP = blood pressure. CI = confidence intervals. CRC = colorectal cancer. FBS = fasting blood sugar. HbA1c = haemoglobin A1C. LDL = low-density lipoprotein.

^a^Complete description of each item available from the authors on request. This column includes the number of participants for whom this CDPS action was assessable at follow-up. Action items intended for women were only assessed for female participants (*n* = 119). ^b^Indicates the patients who were eligible to improve the action at baseline. ^c^Of the patients who were eligible at baseline, indicates the patients who accomplished the action at follow-up.

**Table 4.** Demographic and clinical characteristics associated with differences in composite outcome score

	**Bivariate models**	**Multivariate model^a^**
	**Δ Outcome (95% CI)^b^**	***P* value**	**Δ Outcome (** **95%** **CI)** ^**b**^	***P* value**
**Female**	-7.3 (-13.3 to -1.3)	0.02	-13.8 (-18.9 to -8.7)	<0.001
**Age, years**	0.5 (0.1 to 0.9)	0.02	0.0 (-0.5 to 0.5)	0.99
**Ethnicity:** European	reference	—	reference	—
Indigenous	-0.8 (-10.9 to 9.3)	0.87	-4.9 (-12.2 to 2.5)	0.20
Other	7.1 (-9.4 to 23.7)	0.40	6.1 (-7.0 to 19.1)	0.36
**Citizenship:** Canadian	reference	—	reference	—
Non-Canadian	10.9 (2.4 to 19.4)	0.01	5.3 (-5.3 to 15.9)	0.33
**Education:** ≤High school	reference	—	reference	—
Some college/university	12.1 (3.7 to 20.5)	0.005	13.7 (0.3 to 27.0)	0.04
Graduate degree	18.0 (11.6 to 24.4)	<0.001	21.6 (6.1 to 37.0)	0.006
**Employment:** Fully employed	reference	—	reference	—
Retired	2.8 (-2.5 to 8.2)	0.30	-3.7 (-11.0 to 3.5)	0.31
Other	0.1 (-5.9 to 6.0)	0.98	-2.1 (-9.9 to 5.6)	0.59
**Relationship:** Partnered	reference	—	reference	—
Non-partnered	3.0 (-3.8 to 9.8)	0.39	-2.2 (-9.1 to 4.6)	0.52
**Income:** $0–$60 000	reference	—	reference	—
$60 000–$100 000	-2.3 (-9.0 to 4.5)	0.52	-7.0 (-17.0 to 2.9)	0.16
≥$100 000	-6.1 (-12.4 to 0.1)	0.06	-11.5 (-20.7to -2.4)	0.01
**Smoking status:** Non-smoker	reference	—	reference	—
Smoker	-16.7 (-23.7 to -9.7)	<0.001	-8.8 (-17.5 to -0.1)	0.05
**Alcohol consumption:** Never	reference	—	reference	—
Less than weekly	9.6 (1.9 to 17.4)	0.02	3.4 (-3.5 to 10.3)	0.34
Weekly or more	-1.2 (-6.5 to 4.2)	0.68	-11.5 (-22.3 to -0.6)	0.04
**Exercise:** <150 minutes/week	reference	—	reference	—
>150 minutes/week	-1.3 (-8.5 to 5.9)	0.73	-1.2 (-7.8 to 5.5)	0.73

CI = confidence intervals. ^a^Multivariate models contain all listed variables, plus variables for study site. ^b^ Data are difference in composite outcome relative to the reference condition for the variable (for example, women achieved a mean of 7.3% less than men on the unadjusted analysis, and 13.8% less than men on the composite outcome after adjustment for the effect of other covariates)

## Discussion

### Summary

Together with the results of the BETTER trial, this programme evaluation provides further support that the BETTER Program can be an effective approach to prevention in the real-world setting. The annual physical exam, traditionally used as an approach to deliver preventative care, has not been effective at achieving improvements to health.^13^ The sample in BETTER 2 presented with more health and social challenges, with an average of 12.3 eligible actions per patient, compared with the BETTER trial, where patients were eligible for an average of 8.9 actions. Despite this, the average achievement of prevention and screening targets in the current study (49%; 6.0 actions) was broadly comparable with that from the BETTER RCT (54%; 4.7 actions). In the BETTER 2 implementation study, a control group was not compared to, but the usual care control group in the BETTER trial achieved just 21.1% of eligible actions.^6^ Improvements in screening tests were observed that are relatively easy to achieve (for example, diabetes and hypertension screening), but changes on important items that have a very well-established connection to improved health, such as improvements in low-density lipoprotein cholesterol, blood pressure, physical activity, obesity, smoking rates, and alcohol misuse, were also observed (Table 2).

Multivariate analysis of the results demonstrated a compelling observation that participants were able to make improvements to their lifestyle and screening irrespective of income, sex, or ethnic group. Furthermore, it was found that income was inversely proportional to the improvement in CDPS, and that men were more successful in achieving their CDPS goals than women.

### Strengths and limitations

Although the three BETTER 2 study clinics were selected based on the intention to target more underserved patients than those enrolled in the BETTER trial, the success was modest in this regard. Indigenous patients were recruited from a remote region and patients from a rural clinic were recruited. However, despite including an urban practice, with a high proportion of patients who live in poverty, the patients enrolled from that practice tended to be well-educated, with relatively high incomes (Table 1). The rural clinic site recruited several participants with lower incomes, but this site experienced significant provider turnover during the study period, which negatively affected the recruitment and retention of patients in the study. Interestingly, this same clinic has had a stable provider population for the last few years and has successfully delivered the BETTER intervention to over 60 patients outside of the research setting in the last few months. The qualitative factors affecting implementation and uptake of the programme,^9^ and patients’ perspectives of the intervention,^14^ are explored elsewhere, but these studies did not identify reasons for the difficulties experienced reaching underserved populations with this programme.

Because the primary objective of BETTER 2 was to examine factors affecting implementation and uptake of the programme in diverse clinical settings, it did not include a control group. However, given that these results are similar to those from the BETTER trial, it is reasonable to conclude that some of the observed benefits were a result of the intervention. A second limitation is that items in the composite index included process measures, referral to other CDPS resources, and measures of behaviour change, but these were considered equally in the composite outcome rather than weighted based on their ability to impact health.

### Comparison with existing literature

Several reviews suggest that lifestyle interventions in primary care are of limited success,^15,16^ but results are mixed.^17^ In addition to factors documented in the authors’ prior work,^9,17^ the literature supports specific aspects of BETTER, which are likely critical to the success of the programme. First, the consensus from the psychological literature is that a non-confrontational interviewing style, which includes active listening, assessment of readiness for change, and discussion of reasons for change, increases the likeliness of behaviour change.^10,18,19^ A comprehensive assessment of health risk at baseline and specially trained providers who personalise the approach for patients may also be important for success.^6,17^

The setting and participant sample also appear to play a role in determining the success of these programmes. A previous study found that a lifestyle intervention designed to be delivered as part of a regular primary care provider consultation did not demonstrate the health benefits expected of it,^20^ although this may be owing to the short duration of each visit. Interventions dedicated solely to prevention, including the one described in this article, appear more likely to demonstrate a measurable positive change in patient health practices.^6,17,21–26^ These pre-scheduled prevention visits likely attract a patient population with greater 'readiness for change’^27^ than brief interventions that are offered during a regular visit. Although this introduces a selection bias, it may be wise to limit participation to patients who have a greater commitment to change given the resources needed for a longer intervention. In the BETTER studies, it was found that pre-scheduling the BETTER intervention and having the visits clearly dedicated to prevention were important for success.^6,9^ This may be easiest to accomplish if the PP is not the person most responsible for the patient’s ongoing care and/or if the PP is not trained to diagnose, because there will likely be less of a tendency for acute health issues to monopolise the available appointment time. This is the model usually used in the BETTER research studies. However, it has been observed that both acute and preventative care can be offered by the same provider with careful organisation and by communicating the goals of the appointment clearly to patients.

### Implications for research and practice

In the BETTER studies so far, the PPs have had prior training as registered nurses, licensed practical nurses, dieticians, or nurse practitioners. Although all of these individuals performed well in the role, providers with training in diagnosis and investigation had to be cautious to maintain the focus of the appointment on prevention and screening. Because existing CDPS evidence has been incorporated into the BETTER tools, the PPs do not require prior education or in-depth knowledge of the evidence behind the tools. Thus, in keeping with the desire to maximise the scope of health worker practice, the PP role can be assumed by professionals that do not have education in diagnosis and investigation, such as licensed practical nurses.

This study adds additional support to existing evidence from the BETTER trial,^6^ showing that a multifaceted intervention led by a non-physician prevention practitioner can lead to significant improvements in CDPS. The BETTER approach harmonises existing evidence on disease screening, lifestyle factors, and behaviour change, and has successfully translated it into actionable, evidence-based clinical tools. Together, the BETTER tools can be used to comprehensively assess health risks and influence behaviour change using shared decision-making and brief action planning. Resources have been developed and published to train providers on how to deliver this intervention; interested clinicians will find contact information and further resources on the BETTER Program website.^28^ The BETTER team is now conducting a second pragmatic RCT with an enhanced intervention that includes cancer surveillance.

## References

[1] Campbell-Scherer D, Rogers J, Manca D (2014). Guideline harmonization and implementation plan for the better trial: building on existing tools to improve chronic disease prevention and screening in family practice. CMAJ Open.

[2] Grunfeld E, Manca D, Moineddin R (2013). Improving chronic disease prevention and screening in primary care: results of the better pragmatic cluster randomized controlled trial. BMC Fam Pract.

[3] Manca DP, Greiver M, Carroll JC (2014). Finding a better way: a qualitative study exploring the prevention practitioner intervention to improve chronic disease prevention and screening in family practice. BMC Fam Pract.

[4] Manca DP, Campbell-Scherer D, Aubrey-Bassler K (2015). Developing clinical decision tools to implement chronic disease prevention and screening in primary care: the BETTER 2 program (Building on Existing Tools To improvE chRonic disease prevention and screening in primary care). Implementation Sci.

[5] Gutnick D, Gutnick D, Reims K (2014). Brief action planning to facilitate behavior change and support for self-management. J Clin Outcomes Manag.

[6] Turrell G, Hewitt B, Haynes M (2014). Change in walking for transport: a longitudinal study of the influence of neighbourhood disadvantage and individual-level socioeconomic position in mid-aged adults. Int J Behav Nutr Phys Act.

[7] Alkerwi Ala'a, Vernier C, Sauvageot N (2015). Demographic and socioeconomic disparity in nutrition: application of a novel correlated component regression approach. BMJ Open.

[8] Conklin AI, Forouhi NG, Surtees P (2015). Gender and the double burden of economic and social disadvantages on healthy eating: cross-sectional study of older adults in the EPIC-Norfolk cohort. BMC Public Health.

[9] Turrell G, Hewitt B, Patterson C, Oldenburg B (2003). Measuring socio-economic position in dietary research: is choice of socio-economic indicator important?. Public Health Nutr.

[10] Finger JD, Tylleskär T, Lampert T, Mensink GBM (2013). Dietary behaviour and socioeconomic position: the role of physical activity patterns. PLoS One.

[11] Kris-Etherton P, Eissenstat B, Jaax S (2001). Validation for MEDFICTS, a dietary assessment instrument for evaluating adherence to total and saturated fat recommendations of the National Cholesterol Education Program Step 1 and Step 2 Diets. J Am Diet Assoc.

[12] Taylor AJ, Wong H, Wish K (2003). Validation of the MEDFICTS dietary questionnaire: a clinical tool to assess adherence to American Heart Association dietary fat intake guidelines. Nutr J.

[13] Huisman M, Kunst AE, Mackenbach JP (2005). Inequalities in the prevalence of smoking in the European Union: comparing education and income. Prev Med.

[14] Baruth M, Wilcox S, Sallis JF (2011). Changes in CVD risk factors in the activity counselling trial. Int J Gen Med.

[15] Fleming P, Godwin M (2008). Lifestyle interventions in primary care: systematic review of randomized controlled trials. Can Fam Phys.

[16] Jepson R, Harris F, MacGillivray S (2006). A review of the effectiveness of interventions, approaches and models at individual, community and population level that are aimed at changing health outcomes through changing knowledge, attitudes and behaviour. https://www.nice.org.uk/guidance/ph6/evidence/behaviour-change-review-1-effectiveness-review-pdf-369664525.

[17] Butler CC, Simpson SA, Hood K (2013). Training practitioners to deliver opportunistic multiple behaviour change counselling in primary care: a cluster randomised trial. BMJ.

[18] Bóveda-Fontán J, Barragán-Brun N, Campiñez-Navarro M (2015). Effectiveness of motivational interviewing in patients with dyslipidemia: a randomized cluster trial. BMC Fam Pract.

[19] Tuomilehto J, Lindström J, Eriksson JG (2001). Prevention of type 2 diabetes mellitus by changes in lifestyle among subjects with impaired glucose tolerance. N Engl J Med.

[20] Stuck AE, Moser A, Morf U (2015). Effect of health risk assessment and counselling on health behaviour and survival in older people: a pragmatic randomised trial. PLoS Med.

[21] Abraham C, Michie S (2008). A taxonomy of behavior change techniques used in interventions. Health Psychol.

[22] Lambert MJ (2003). Bergin and Garfield’s Handbook of Psychotherapy and Behaviour Change.

[23] Blomstrand A, Ariai N, Baar A-C (2012). Implementation of a low-budget, lifestyle-improvement method in an ordinary primary healthcare setting: a stepwise intervention study. BMJ Open.

[24] Lindström J, Louheranta A, Mannelin M (2003). The Finnish diabetes prevention study (Dps): lifestyle intervention and 3-year results on diet and physical activity. Diabetes Care.

[25] Knowler WC, Barrett-Connor E, Fowler SE (2002). Reduction in the incidence of type 2 diabetes with lifestyle intervention or metformin. N Engl J Med.

[26] Ross R, Lam M, Blair SN (2012). Trial of prevention and reduction of obesity through active living in clinical settings: a randomized controlled trial. Arch Intern Med.

[27] Prochaska JO, DiClemente CC, Norcross JC (1992). In search of how people change. applications to addictive behaviors. Am Psychol.

[28] University of Toronto, University of Alberta and Memorial University (2015). The BETTER Program. www.better-program.ca.

